# Association of Model-Predicted Epigenetic Age and Female Infertility

**DOI:** 10.3390/epigenomes9020019

**Published:** 2025-06-05

**Authors:** Elena Pozdysheva, Vitaly Korchagin, Tatiana Rumyantseva, Daria Ogneva, Vera Zhivotova, Irina Gaponova, Konstantin Mironov, Vasily Akimkin

**Affiliations:** 1 Central Research Institute of Epidemiology Federal Service for Surveillance on Consumer Rights Protection and Human Wellbeing of Russian Federation, Moscow 111123, Russia; 2 Fomin Clinic, Moscow 119192, Russia

**Keywords:** DNA methylation, CpG sites, pyrosequencing, aging, age prediction, infertility

## Abstract

Background: To date, there are no precise clinical and laboratory methods to accurately predict the onset of fertility decline in women, with chronological age being the ultimate predictor. This has led to increased interest in developing methods to determine biological age, as it provides a more accurate understanding of individual age-related physiological changes. Methods: In this study, we developed a model for estimating biological age based on DNA methylation levels in the *ELOVL2*, *TRIM59*, *C1orf132*, *FHL2*, and *KLF14* genes using pyrosequencing. The model was tested in 64 Russian women, aged 25–39 years, to find an association between epigenetic age, infertility, low anti-Müllerian hormone (AMH) levels, and assisted reproductive technology (ART) failure. Results: The predictive performance of the model was evaluated. The mean absolute deviation of the model was 2.8 years; the mean absolute error was 2.6 years (R^2^ = 0.95). In the studied cohort, 33% of women exhibited epigenetic age acceleration (EAA), while 45% showed epigenetic age deceleration (EAD). All women with an EAA of ≥3 years (n = 6) had a history of infertility. Conclusions: In this study, no statistically significant associations were observed between EAA/EAD and AMH, body mass index, infertility, or ART failure in women.

## 1. Introduction

The prevalence of infertility in developed countries is currently estimated to range from 10% to 12% [1]. Various risk factors for infertility have been reported in the literature, including obesity, dietary habits, smoking, alcohol consumption, and aging [2,3,4]. Notably, an increasing number of couples are postponing parenthood until a later age, emphasizing the need to understand how age affects fertility [5].

The concept of biological age and the terms “epigenetic age acceleration” (EAA), when biological age is greater than chronological age, and “epigenetic age deceleration” (EAD), when biological age is less than chronological age, have recently become subjects of considerable interest. This is due to the ability of biological age to more accurately reflect the body’s physiological state than that of chronological age. The epigenetic biomarkers of aging have proven to be reliable predictors of biological aging and the risk of age-related conditions such as cancer, heart disease, neurological disorders, and overall mortality [6]. Despite having the same chronological age, individuals with EAA face higher risks of developing the aforementioned conditions [7].

DNA methylation is a common and stable DNA modification in the context of the long-term storage of biological samples. It strongly correlates with age-related changes (r > 0.8) [8]. Currently, various models exist for estimating biological age based on hypo- and hypermethylation in different regions of the genome and in different tissues. Each model displays its own strengths and weaknesses. Therefore, when conducting studies based on epigenetic age-related changes, it is important to refer to the specific model used, and to calibrate it using a testing group for the most accurate prediction. The statistical methodology used should also be considered [6].

One of the most popular methods for estimating biological age is the Horvath clock [9]. However, while it analyzes a large number of CpG sites, it is relatively expensive and may therefore not be appropriate for all research settings due to the costs involved and the complexity of the analysis. In everyday practice, models based on a limited number of CpG sites are of the greatest interest, as they are easy to implement and cost-effective, yet highly accurate. Such types of models have been proposed by Zbieć-Piekarska et al. [10,11] and Park et al. [12]. These models demonstrate high levels of prediction accuracy, as evidenced by a mean absolute deviation (MAD) from chronological age of less than 4 years. The models proposed by Zbieć-Piekarska et al. [10,11] are based on the results of measuring the methylation level of CpG sites in the *KLF14*, *FHL2*, *TRIM59*, *C1orf132*, and *ELOVL2* genes using pyrosequencing. In their studies, DNA methylation analysis was performed on 425 samples and 427 samples, respectively [10,11]. The age prediction efficiency of this model was subsequently confirmed in various studies involving different samples: in 100 Korean blood samples [13], in 190 peripheral blood samples from patients with Graves’ disease and Alzheimer’s disease [14], and in 176 athletes compared to 128 healthy control individuals from the general population [15]. Currently, the *KLF14*, *FHL2*, *TRIM59*, *C1orf132*, and *ELOVL2* genes are among the most commonly used genes for estimating biological age in methylation analysis [16]. In the present study, we attempted to evaluate the possibility of using CpG sites from the Zbieć-Piekarska et al. models [10,11] to create an age-prediction model for the Russian population.

A number of studies have demonstrated a correlation between biological age and female fertility [17]. Although, to date, the exact causal relationship between epigenetic age-related changes and infertility has not yet been precisely established, it can be hypothesized that the internal processes leading to a decline in or the absence of, reproductive capabilities may be an indirect measure of biological aging. This finding is consistent with studies showing that the premature loss of ovarian function caused by a bilateral ovariectomy performed before the onset of natural menopause increases the susceptibility to age-related conditions [18]. Transplanting young ovaries into old mice has also been shown to significantly increase lifespan [19]. Research suggests that menopausal hormone therapies, which counteract some of the effects of menopause, are associated with decreased epigenetic age [20]. In addition to the association with biological age, several studies of the aforementioned genes used in the Zbieć-Piekarska et al. model [10,11] have found associations between polymorphisms, gene expression, and various pathologies, including metabolic and cardiovascular diseases, as well as with factors affecting fertility and placental and fetal growth [21,22,23,24,25,26,27,28,29,30,31,32,33,34,35,36,37,38,39,40]. In the context of the association between EAA and reduced fertility, infertility, and pregnancy problems, analyzing these age-associated markers is of interest.

This study aimed to develop a model for calculating biological age based on DNA methylation levels (*KLF14*, *FHL2*, *TRIM59*, *C1orf132*, and *ELOVL2)* using the pyrosequencing technique. The model was tested using a sample of women diagnosed with infertility and with a history of perinatal loss. The study also aimed to identify potential associations between EAA/EAD and infertility, as well as with low anti-Müllerian hormone (AMH) levels (<1.2 ng/mL), an abnormal body mass index (BMI) (<18.5 or >25), and assisted reproductive technology (ART) failure.

## 2. Results

### 2.1. Selection of CpG Sites for Biological Age Prediction

Statistically significant correlations of methylation levels with chronological age were found for all six CpG sites in the training group. The strength of these correlations ranged from moderate (0.5–0.7) for *TRIM59* to strong (0.7–0.9) and very strong (0.9–1.0) for the remaining genes (Table 1).

A high correlation was observed between methylation levels and age in the training group for all analyzed CpG sites. A linear trend of methylation level increase was observed for *KLF14*, *TRIM59*, and *ELOVL2* C7 up to 40 years of age, for *FHL2* up to 50 years of age, and for *ELOVL2* C5 and *C1orf132* only up to 70 years of age. An opposite trend was observed in the 80-year-old group (Table 2).

**Figure 1:**
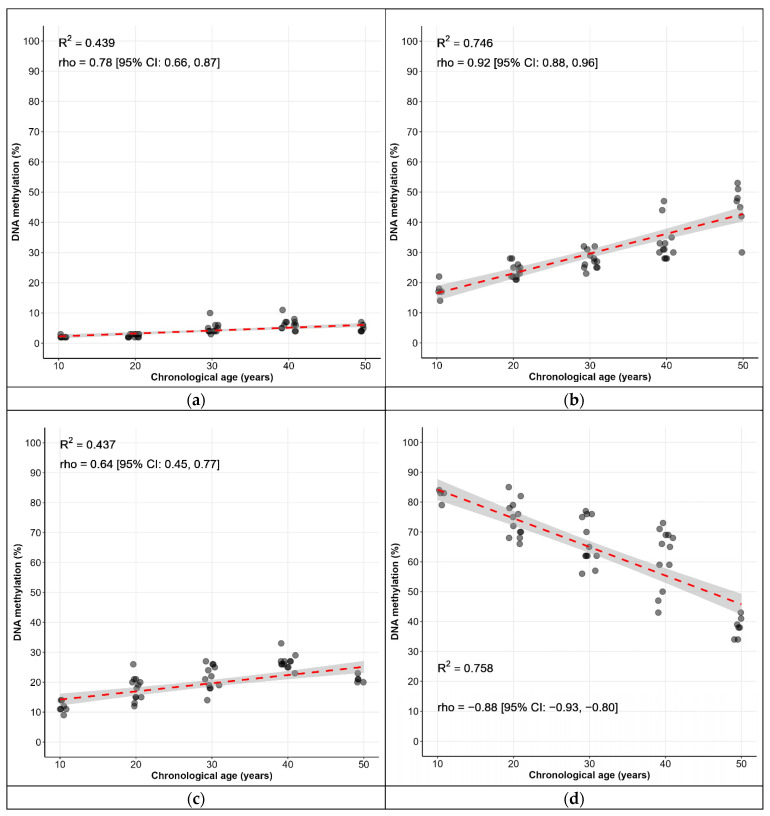

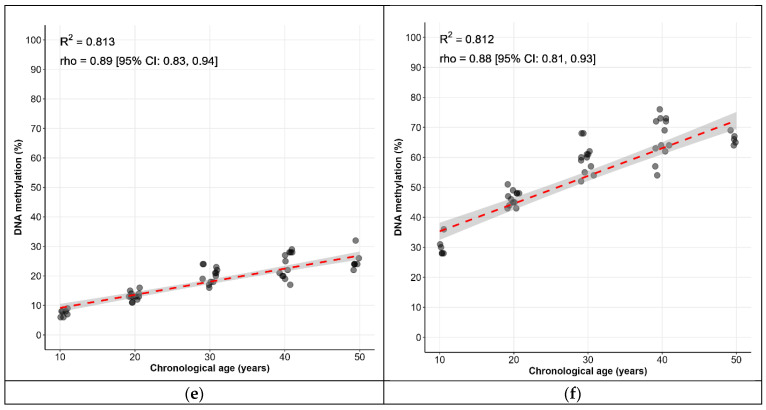
Correlation between chronological age and methylation at six CpG sites: (**a**) *KLF14* C1, (**b**) *FHL2* C2, (**c**) *TRIM59* C7, (**d**) *C1orf132* C1, (**e**) *ELOVL2* C5, and (**f**) *ELOVL2* C7 in 60 samples from the training groups before age 50. Each dot corresponds to a single sample. Darker dots are the result of the complete overlap of some dots. The red dashed lines with a shaded area correspond to the regression lines, with a 95% confidence interval. Each panel reports the coefficient of determination (R^2^) and the Spearman’s correlation coefficient (rho).

In general, the differences in the methylation levels of the analyzed genes were limited to the age subgroups of 10–50 years. In addition, the methylation data for the subgroups over 50 years old for *FHL2*, *TRIM59*, and *ELOVL2* C5, and for the subgroup over 30 years old in the case of *KLF14*, were characterized by a large data variation and significantly overlapping distributions (Figure S1). Based on these results, we assumed that including patients over 50 years old in the prediction model might reduce its quality. In addition, in the female test cohort, the age range was restricted to 40 years. Consequently, individuals in the training group who were over 50 years of age were excluded from the analysis. Within the range of 10–50 years, the variations in CpG methylation levels show a more pronounced linear trend, with a strong correlation between methylation levels and age. In some cases, there is an increasing correlation (Figure 1).

### 2.2. Biological Age Prediction Model

DNA methylation data from all six CpG sites in *KLF14*, *FHL2*, *TRIM59*, *C1orf132* and *ELOVL2* were selected to create an initial age prediction model (named Age_predict1) using multivariate linear regression with 10-fold cross-validation. In the second model (named Age_predict2), only informative loci selected in WEKA [41] using the M5’s method were included. The key features of both age prediction models are summarized in Table 3. Since the ANOVA results in a *p* = 0.937, we cannot reject the null hypothesis and conclude that the Age_predict1 model provides a significantly better fit than the Age_predict2 model. Both models exhibit close correlation coefficients with chronological age, root mean squared error (RMSE), and mean absolute error (MAE). Thus, the inclusion of the *TRIM59* methylation level in the model does not affect its prognostic qualities. The prediction model with fewer independent variables that display equal or slightly different characteristics has a lower Akaike criterion value and is preferable for use.

### 2.3. Testing of a Biological Age Calculation Model Using a Sample of Women with Perinatal Loss and Infertility

**Figure 2:**
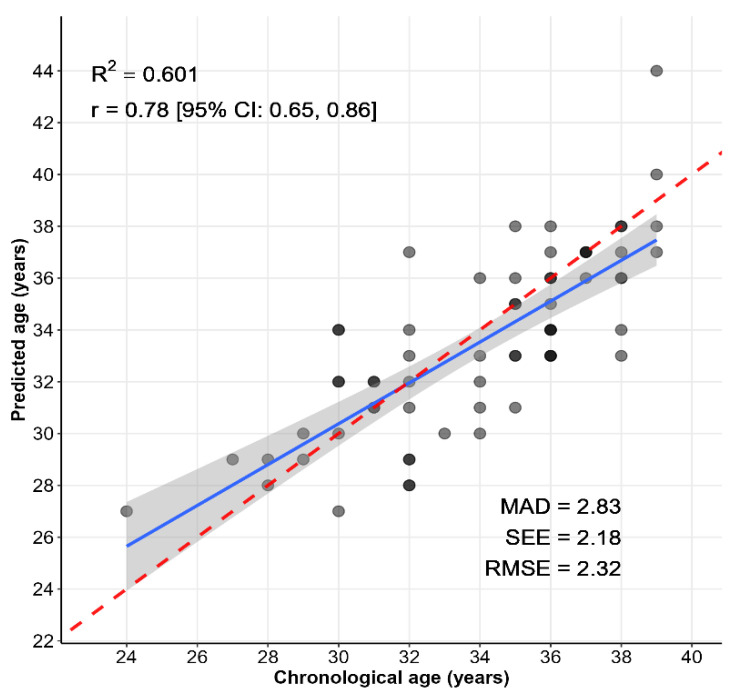
Correlation between chronological age and age predicted using the Age_predict2 model in the testing group. MAD, mean absolute deviation; SEE, standard error of the estimate; RMSE, root mean squared error. Darker dots are the result of the complete overlap of some dots. The red dashed and blue lines correspond to the diagonal line (*y* = *x*) and the regression line (with the 95% confidence interval as shaded area), respectively.

We evaluated the Age_predict2 model performance with the testing set and observed that the RMSE, standard error of the estimate (SEE), and MAD were 2.32, 2.18, and 2.83, respectively (Figure 2). Samples with correctly predicted ages shown are on the red dashed line and account for 23% of the samples.

In the 64 women studied, the mean chronological age was 33.9 years (SD = 3.4), and the mean biological age was 33.5 years (SD = 3.5). Among all patients, 25% had a BMI below or above normal, and 27% exhibited low AMH levels. The most common primary diagnoses were infertility of unknown etiology (44%), habitual non-pregnancy (19%), and recurrent implantation failure (RIF) (19%). Diagnoses were not mutually exclusive. Most patients participated in different ART programs (64%, n = 41), while 71% of patients (n = 29) achieved a successful pregnancy and delivery.

The patients were divided into four groups, according to their medical history and ART results (Table 4). Group I included healthy women with no history of pregnancy problems and at least one live birth (n = 7). Women with a history of infertility or habitual non-pregnancy represented groups II-IV. Group II comprised patients who had never participated in ART (n = 16). Group III included patients who had participated in different ART programs and achieved pregnancy with subsequent delivery (n = 29). Group IV included patients who participated in different ART programs but did not achieve pregnancy (n = 12).

**Figure 3:**
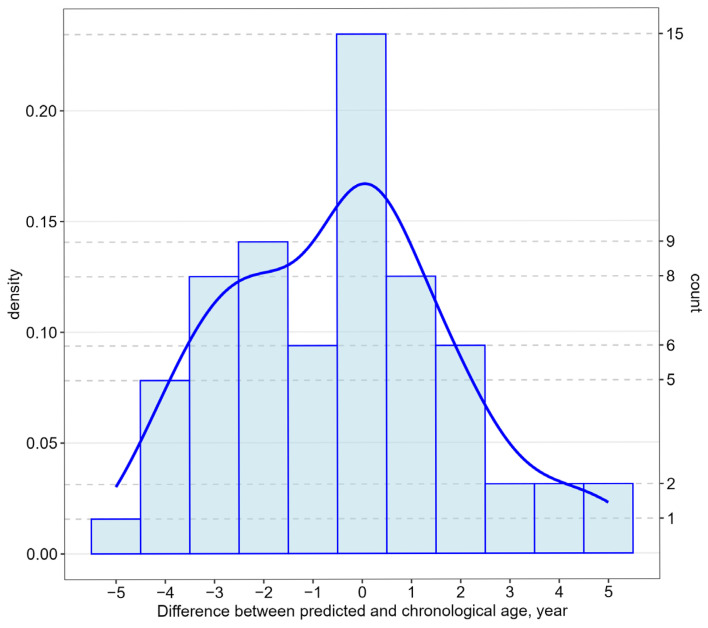
Distribution of patients from the testing group by difference of predicted age from chronological age.

The data range of age acceleration/deceleration discrepancy was from 1 to 5 years (Figure 3). EAD was observed in 45% of the patients (n = 29), and EAA was observed in 33% of the patients (n = 21). In all patient groups studied, the chronological age and the biological age were not statistically different (*p* > 0.05) (Table 5).

However, it should be noted that, despite the lack of statistical significance between the groups, all patients with EAA ≥ 3 years (n = 6) had a history of infertility (one woman in Group II and five women in Group III). Four patients exhibited infertility of unknown etiology, and two had a history of habitual non-pregnancy. Two patients were also diagnosed with endometriosis, and one showed diminished ovarian reserve. Three out of five patients participated in ART programs more than once. Ultimately, all five patients achieved pregnancy and subsequent delivery.

The association between EAA/EAD and reduced ovarian reserve was studied. No difference was observed in either chronological or biological age between patients with AMH < 1.2 ng/mL and those with AMH > 1.2 ng/mL. Additionally, the low AMH group was biologically younger than their chronological age (33.30 years vs. 35.00 years, *p* = 0.08) and exhibited a small EAD (∆ = 1.70) (Table 5).

The study found that biological age was unaffected by being underweight or overweight. Patients with a normal BMI and patients with an abnormal BMI were biologically younger in both groups (Table 5).

The association between biological age and women’s participation in ART, with different outcomes, was also examined. Patients who had participated in such programs were found to be slightly biologically younger than their chronological age (33.30 years compared with 35.00 years, *p* = 0.19). Conversely, patients without a history of ART exhibited a biological age that was more advanced than their chronological age (33.20 years vs. 33.00 years, *p* = 0.37) (Table 5).

There was no association between age and ART outcomes in either Group III (no pregnancy) or Group IV (pregnancy). Both groups were biologically younger than their actual age (*p* = 0.30 and *p* = 0.56, respectively) (Table 5).

A control group of healthy women without perinatal loss or infertility was compared with a group of patients whose ART protocols had failed (Groups I and IV). The two groups were found to be biologically younger (*p* = 0.67 and *p* = 0.56, respectively) (Table 5).

In all cases, the EAA/EAD date was not statistically significant (Table 5).

## 3. Discussion

Reproductive aging is a natural process that occurs in women and ultimately results in menopause. According to the literature, approximately 10% of women experience early menopause, characterized by an accelerated decline in fertility from around the age of 32, difficulty conceiving at a relatively young age, and the onset of menopause by the age of 40–45 [42].

Research findings indicate that infertility can substantially impact a women’s quality of life. This is evidenced by its association with elevated rates of low self-esteem, depression and increased mortality rates [43]. Despite the existence of various markers for assessing ovarian reserve and fertility, there is currently a lack of optimal clinical and laboratory methods capable of identifying women experiencing early reproductive aging. Follicle-stimulating hormone (FSH), estradiol (E2), antral follicle count (AFC), and AMH are frequently used as markers to assess ovarian reserve. However, these markers lack the capacity to predict the onset of a significant decline in fertility and are more indicative of the ovarian response during ART than of the ability to conceive naturally [42].

A number of studies have now demonstrated an association between EAA and fertility decline and ART outcomes [17]. In this study, we sought to construct and examine an age prediction model using DNA methylation data from multiple loci [10,11]. These loci have been demonstrated to display satisfactory predictive capabilities in independent research studies [44]. The model was evaluated using a cohort of women in the Moscow region.

For five of the six loci, the correlation between methylation levels and chronological age was quite high in the training group, and the relationship was linear over the entire age range from 10 to 50 years. For the four sites in the over-50 age group, a non-linear dependence of methylation levels on age was observed, and the accuracy of age prediction in this age group could be lower than that for younger patients. This is consistent with the Zbieć-Piekarska model [11], which tends to underpredict age in the elderly [44]. Although the *TRIM59* was incorporated into the age prediction models in previous studies [11,45], it was excluded from our study due to its non-significant effect on model performance.

We examined the association between EAA/EAD and low AMH levels, as well as various ART outcomes, in a sample of Russian women of reproductive age. However, we did not find any associations with infertility diagnosis, AMH levels, various ART outcomes, and BMI, which is inconsistent with the results of previous research [46,47]. In contrast, Monseur et al. [27] reported an association between EAA and low AMH levels (1.29 vs. 2.29 ng/mL, *p* = 0.05) and lower AFC (8 vs. 14.5, *p* = 0.05) in women undergoing ART.

As expected, the epigenetic age exhibited a robust correlation with the chronological age in this study. Contrary to the findings reported in Ref. [47], however, no significant disparities were observed between women with AMH levels below 1.2 ng/mL and those with AMH levels above 1.2 ng/mL. Potential explanations for these discrepancies may include the limited sample size, and the fact that, while AMH exhibits an inverse correlation with chronological age, its values can be influenced by a multitude of reproductive, environmental, and lifestyle factors that were not considered in this study [48].

It can also be hypothesized that the absence of a correlation between EAA/EAD and the range of diagnoses and outcomes associated with ART use is due to population factors. To the best of our knowledge, no study of this nature has been conducted among Russian women. Most studies investigating the correlation between epigenetic aging, various diseases, and environmental exposures have been replicated in different populations. However, a number of studies have demonstrated that some CpG sites used to assess external factors (e.g., smoking) are not specific to all ethnic groups [49,50]. In a study by Robert Philibert et al., methylation status at 50 of 513 CpG sites regarding DNAm PhenoAge (Levin clock) was associated with ethnicity (*p* < 0.05), which may lead to inconsistent results across samples of different races/ethnicities when examining association of EAA with health behaviors. The authors concluded that several methylated sites used in the epigenetic clock require adjustment for ethnicity [51]. There is a possibility that the sample we studied also contains unknown distinctive demographic characteristics. This hypothesis requires validation; therefore, it is necessary to expand the sample and compare it with results for other ethnic groups residing in Russia.

The discrepancies observed between the findings of the studies can be attributed to the non-specific nature of epigenetic effects, which exhibit significant variability due to the influence of additional factors. In the present study, we were only able to assess the influence of BMI due to a lack of information on other potential factors. Published studies demonstrate that smoking, diet, education level, comorbidities such as type 2 diabetes mellitus, and infectious diseases affect methylation levels [52,53,54,55,56,57].

We recognize that the limitations of our research are inherent in the use of a limited number of CpG sites, which may have resulted in an underestimation or overestimation of biological age. However, we consciously aimed to create an easy-to-use predictive model. The studies examining the associations between biological aging and various diseases use next-generation models to predict biological age. These models are based on the machine selection of the most informative CpG sites (from large genome-wide methylation datasets) that are associated with non-age-related factors, such as various conditions, lifestyles, and diseases [9,52,58,59,60,61]. While methylation data from the *ELOVL2*, *TRIM59*, *C1orf132*, *FHL2*, and *KLF14* are frequently used in epigenetic clocks [62], the Zbieć-Piekarska R. et al. model [10,11], from which the CpG sites for this study were derived, is predominantly used in forensic practice to determine chronological age. However, a notable exception is observed in a study by Spolnicka et al., in which this model exhibited a substantial EAA in patients with early Alzheimer’s disease [14].

It has been demonstrated that our model displays reasonably good age prediction accuracy compared to that of some of the most commonly used models based on DNA methylation datasets (MAE = 2.6; RMSE = 3.6; r = 0.97 vs. Horvath: r = 0.89; MAE = 1.2 and Hannum: r = 0.9; RMSE = 4.89) [8,59]. However, it should be noted that our design may have overlooked the influence of some CpG sites that are more influenced by biological indicators than chronological age. Due to the limited number of individuals included in our study, our conclusions about methylation-based age prediction model accuracy should be further confirmed by other validation studies using larger cohorts.

Genetic polymorphisms and epigenetic modifications play an important role in ovarian function, pregnancy progression, and placental and fetal growth. However, further research is required to ascertain the precise function of age-related epigenetic biomarkers and their potential for predicting fertility or the effectiveness of ART. In our study, we sought to investigate the possible involvement of the *ELOVL2*, *TRIM59*, *C1orf132*, *FHL2*, and *KLF14* genes in reproductive aging, infertility, and outcomes of ART.

The *KLF14* gene is a key metabolic transcriptional transregulator with monoallelic maternal expression that mediates adipogenesis, insulin signaling, lipid metabolism, and inflammatory and immune responses, as well as cell proliferation and differentiation [21]. A number of studies have revealed an association between *KLF14* polymorphisms and methylation status, which were associated with lipid profiles, blood pressure status, insulin resistance markers, and metabolic syndrome [22]. Hormonal disruptions, metabolic syndrome, and epigenetic modifications in obesity may adversely affect reproductive health and the success rates of ART and pregnancy outcomes. Interestingly, experiments in mice show that loss of the maternal *KLF14* locus in the mouse placenta results in changes to the gene expression patterns that modulate placental growth [23].

In the context of aging markers, the methylation levels of the CpG sites within the promoter region of the very long chain fatty acid elongase 2 gene *ELOVL2*, coding for a transmembrane protein involved in the synthesis of long omega-3 and omega-6 polyunsaturated fatty acids, have been linked to chronological age across diverse populations, cell types, and tissues [24,25,26]. The methylation of *ELOVL2* accelerates aging in the mouse retina [27,28] and has been linked to an increased risk of breast and colorectal cancer [29]. Alterations in the *ELOVL2* methylation status have been observed in patients in the early stages of Alzheimer’s disease [14]. Furthermore, changes in *ELOVL2* expression are involved in the synthesis of long-chain polyunsaturated fatty acids required for fetal growth during pregnancy [30].

*FHL2* is a key regulator of intracellular signal transduction pathways and contains a number of methylation-prone CpG sites that have repeatedly been shown to become methylated with age, thereby regulating *FHL2* expression [31,32]. *FHL2* is most abundantly expressed in the heart and ovaries. Remarkably, *FHL2* expression increases with methylation [33]. The *FHL2* genetic polymorphisms are associated with various metabolic, cardiovascular, and oncological diseases and traits, which are predominantly age-related. For instance, rs114298934 has been associated with age of menopause, while rs3087523 and rs186607487 have been associated with BMI and fat body mass, respectively [34,35,36].

*C1orf132* (also known as *MIR29B2CHG*) is an intergenic lncRNA located on 1q32.2 between the CD34 and CD46 protein coding genes. *C1orf132* is the host gene for miR-29b2 and miR-29c; these miRNAs exhibit both tumor suppressive and oncogenic roles in different cancers. The *C1orf132* promoter is most often hypermethylated in basal-like breast tumors. However, *C1orf132* also expresses longer transcripts, whose functions remain unknown [37].

The human tripartite motif (TRIM) protein family has been implicated in a wide range of physiological processes, including immunity, proliferation, antiviral processes, oncogenesis, and transcriptional regulation. It is also involved in various biological processes, such as cellular growth, metastasis, and development. Tripartite motif-containing 59 (TRIM59), a surface molecule belonging to the TRIM family, plays a role in tumor proliferation and migration [38]. However, overexpression of TRIM59 is also observed in endometriosis, with TRIM59 promoting the invasion of ectopic endometrial stromal cells [39]. Decreased TRIM59 expression in mouse spermatogonial cells impairs their viability, migration, and proliferation and increases apoptosis, which may be one of the factors affecting male fertility [40]. Currently, chronological age remains the most significant predictor of reproductive decline. This study did not demonstrate sufficient data regarding the association and feasibility of using predicted biological age to identify patients at increased risk of early reproductive aging, infertility, or ART failure. Further research is necessary to address this knowledge gap. The discovery of new molecular candidates that are sufficiently accurate in detecting biological age-related changes with non-invasive, inexpensive, accessible, and rapidly interpretable testing could improve the diagnostic evaluation of ovarian reserve and decline in fertility.

## 4. Materials and Methods

### 4.1. Ethics Approval

The study was approved by the Ethics Committee of the Central Research Institute of the Epidemiology Federal Service for Surveillance on Consumer Rights Protection and Human Wellbeing, Russian Federation (protocol number 116, 29 June 2021), and was conducted in accordance with the Declaration of Helsinki. All participants signed informed consent statements prior to sample collection.

### 4.2. The Study Population and Blood Collection

A training group of 94 DNA samples, isolated from the whole blood of depersonalized patients aged between 10 and 80 years and taken from the of the Central Research Institute of Epidemiology biobank, was used to develop the epigenetic clock model. Over 90% of the training group are residents of the Moscow region, with Caucasian ancestry.

In addition, this study involved 64 women, aged 24 to 39, who visited the Fomin Clinic, a multidisciplinary medical center in Moscow. Blood samples were collected from these individuals in order to assess the association between infertility, ART outcomes, and biological age. A total of 57 out of 64 women reported reproductive problems: infertility (primary infertility (n = 12), unexplained infertility (n = 19)), uterine myoma (n = 11), endometriosis (n = 13), habitual non-pregnancy (n = 11), and diminished ovarian reserve (n = 17). These diagnoses were not mutually exclusive. Of the 57 women, 41 had previously participated in ART programs, with varying outcomes: 29 achieved pregnancy and subsequent successful delivery, while 12 did not. Some of the patients participated in ART programs more than once (n = 14). The inclusion criteria for women with failed ART protocols comprised the lack of implantation after the transfer of at least one euploid embryo or an untested three- to five-day embryo of good quality. An additional seven healthy women, who had no reproductive problems and had given birth at least once before, were included in the study as the control group. All diagnoses were made by clinicians, in accordance with medical standards and the International Classification of Diseases. The demographic composition of the study sample is as follows: 95% of the participants were of Russian origin. The clinical characteristics of the participants, including age, BMI, AMH levels, and group distribution based on anamnesis, are outlined in Table 4. To ensure that the most recent measurements were collected, AMH values were obtained in close proximity to the time of blood collection for the study.

### 4.3. DNA Extraction

The isolation of DNA from whole blood samples was performed using the RIBO-Prep kit (AmpliSens, Moscow, Russia), in according with the manufacturer’s protocol. Subsequent storage of the isolated DNA was conducted at −20 °C until its utilization. The quantification of the isolated DNA was analyzed using real-time PCR, employing primers specific to the β-globin gene with reagents from the Central Research Institute of Epidemiology and RotorGene instrumentation (Qiagen, Hilden, Germany).

### 4.4. Bisulfite Conversion

The EpiTest Bisulfite kit (200) (Qiagen, Hilden, Germany) was used for the bisulfite conversion of DNA. Genomic DNA samples (200 ng) were incubated with the modifying reagent for 12 h at 56 °C. The volume of the reaction mixture was 200 µL. The use of such an incubation protocol has been shown to reduce the degradation of DNA during conversion, thus enhancing the integrity of the subsequent pyrosequencing data and consequently reducing the number of permutations. Following incubation, purification was achieved by following the kit’s instructions. Subsequent to bisulfite conversion, the samples were not stored, but rather PCR was conducted immediately.

### 4.5. PCR and Pyrosequencing

Five genes (*ELOVL2*, *TRIM59*, *C1orf132*, *FHL2*, *KLF14*) were selected as targets for methylation analysis (Table 6) [10,11].

The methylation levels were measured using pyrosequencing. For pyrosequencing, forward, reverse, and sequencing primers were designed with PyroMark Assay Design Software v2.0 (Qiagen, Hilden, Germany). PCR, sequencing primer sequences, and the analysis sequence are given in Supplementary Table S1. The PCR reaction was performed in a 25 μL tube containing 10 μL of bisulfite-converted DNA, 4.5 μL of PCR buffer (AmpliSens, Moscow, Russia), 0.5-μL of TaqF polymerase (AmpliSens, Moscow, Russia), 0.88 mM dNTP, and 10 pmol of each primer. All PCR reactions were performed with an annealing temperature of 59 °C for 45 cycles. The PCR product (5–15 μL) was prepared for subsequent analysis using the Pyro-Prep Reagent Kit (Amplisens, Moscow, Russia). The sequencing was performed on the PyroMark Q24 using the Pyro Gold Reagent Kit (Qiagen, Hilden, Germany), according to the manufacturer’s instructions. The resulting pyrograms were analyzed using PyroMark Q24 v2.0.8 software. The methylation levels in the CpG sites of the *ELOVL2*, *TRIM59*, *C1orf132*, *FHL2*, and *KLF14* genes for the training and test groups are given in Supplementary Tables S2 and S3.

### 4.6. Statistical Analysis

All statistical analyses and data processing steps were performed using MS Excel (Microsoft) and R version 4.4.1 [63]. The Spearman’s correlation was used to analyze the association between DNA methylation and aging in the training group of 94 subjects ranging in age from 10 to 80 years. For the between-group comparisons, we used a non-parametric Kruskal–Wallis test and a Mann–Whitney U test. A *p*-value of <0.05 was considered statistically significant. The results of the statistical analysis were visualized using the ggplot2 and ggstatsplot packages [64,65].

### 4.7. Development of the Age Prediction Model

To develop of the age prediction model, we used the linear regression classification method with the default parameters and a 10-fold cross-validation using WEKA Workbench (version 3.8.5) [41]. The correlation between actual age and predicted age was assessed using the Pearson’s r correlation coefficient and the coefficient of determination R². For the age prediction model, the MAD, SEE, and RMSE were calculated. RMSE was calculated as RMSE = mean((observed−predicted)^2^). The lower the RMSE, the better the model. To compare the models, we used ANOVA and functions from the performance package [66].

## Figures and Tables

**Table 1:** Correlation between DNA methylation levels and age in the training group.

Locus	CpG Sites	Spearman’s *ρ* [95% CI] *
*KLF14*	C1	0.77 [0.67, 0.84]
*FHL2*	C2	0.85 [0.79, 0.90]
*TRIM59*	C7	0.60 [0.45, 0.72]
*C1orf132*	C1	−0.92 [−0.95, −0.88]
*ELOVL2*	C5	0.94 [0.91, 0.96]
	C7	0.88 [0.82, 0.92]

* *p* < 0.0001 in all cases.

**Table 2:** DNA methylation levels in age subgroups of the training group.

Age Group, Year (n)	Methylation %, Mean (SD) *					
	*KLF14*	*FHL2*	*TRIM59*	*C1orf132*	*ELOVL2* C5	*ELOVL2* C7
10 (12)	2.17 (0.39)	17.7 (2.46)	11.4 (1.62)	82.2 (2.52)	8.08 (1.51)	30.1 (2.43)
20 (12)	2.50 (0.52) ↑	23.9 (2.50) ↑	17.9 (4.03) ↑	74.1 (6.05) ↓	13.2 (1.47) ↑	46.4 (2.50) ↑
30 (12)	4.92 (1.83) ↑	27.5 (2.97) ↑	21.6 (4.08) ↑	66.7 (7.74) ↓	20.2 (2.70) ↑	59.8 (4.94) ↑
40 (12)	6.33 (1.92) ↑	33.2 (6.19) ↑	26.8 (2.45) ↑	61.6 (10.0) ↓	23.7 (4.27) ↑	66.6 (6.96) ↑
50 (12)	**5.00 (1.04) ↓**	45.8 (8.53) ↑	**20.7 (2.77) ↓**	40.4 (6.08) ↓	25.0 (2.63) ↑	**66.2 (1.99) ↓**
60 (12)	6.50 (1.24) ↑	**37.8 (4.45) ↓**	23.5 (3.29) ↑	38.1 (6.04) ↓	30.7 (3.23) ↑	72.8 (3.16) ↑
70 (12)	7.58 (2.43) ↑	45.8 (7.19) ↑	26.1 (10.4) ↑	28.7 (10.1) ↓	37.6 (8.37) ↑	75.6 (3.55) ↑
80 (10)	7.70 (2.21) ↑	46.6 (5.10) ↑	30.1 (6.06) ↑	**29.2 (7.15) ↑**	**37.0 (5.25) ↓**	**72.1 (2.81) ↓**

* Arrows indicate the direction of methylation level changes relative to the previous value. Methylation levels opposite to the age-related linear trend are shown in bold and fill color.

**Table 3:** Main characteristics of age prediction models.

Name	Model Equation	SE	Statistical Summary
Age_predict1	Age = 24.4506 + 0.1492 × *FHL2* + 0.4488 × *KLF14* + 0.0114 × *TRIM59* + −0.3771 × *C1orf132* + 0.3328 × *ELOVL2* C5 + 0.3259 × *ELOVL2* C7	5.727 0.0833 0.3094 0.1432 0.0514 0.2235 0.1148	R^2^: 0.954 R^2^_Adjusted_: 0.9488 Correlation coefficient: 0.9674 MAE: 2.6158 RMSE: 3.5943 Relative absolute error: 21.42% Root relative squared error: 25.17% AIC: 319
Age_predict2	Age = 24.3118 + 0.1495 × *FHL2* + 0.4492 × *KLF14* + −0.3755 × *C1orf132* + 0.3335 × *ELOVL2* C5 + 0.3303 × *ELOVL2* C7	5.4046 0.0825 0.3065 0.047 0.2212 0.0996	R^2^: 0.954 R^2^_Adjusted_: 0.94975 Correlation coefficient: 0.9675 MAE: 2.6039 RMSE: 3.5866 Relative absolute error: 21.33% Root relative squared error: 25.12% AIC: 317

The color indicates the regression model member removed in Age_predict2 model.

**Table 4:** The clinical characteristics of the studied sample of women (n = 64).

Group	n	Age, y (SD)	BMI, kg/m^2^ (SD)	AMH, ng/mL (SD)
Group I. Control group: healthy individuals who have had at least one live birth and have no history of perinatal losses or infertility diagnoses.	7	33.3 (3.5)	21 (3.7)	5.2 (3.2)
Group II. Women with a history of infertility diagnosis or perinatal losses; ART was not applied.	16	33.4 (3.4)	21 (1.5)	3.6 (4.1)
Group III. Women experiencing infertility or perinatal losses and have undergone ART and achieved successful pregnancies, resulting in delivery.	29	34.1 (3.6)	22 (3.3)	2.1 (1.5)
Group IV. Women experiencing infertility, perinatal losses, and assisted reproductive technology (ART), without pregnancy occurrence.	12	34.6 (3.1)	22 (4.2)	3.4 (1.4)
	n (%)			
Age:				
24–29	6 (9%)			
30–34	25 (39%)			
35–39	33 (52%)			
BMI abnormal (>18.5 or <25)	16 (25%)			
AMH < 1.2 ng/mL	17 (27%)			

**Table 5:** Comparative analysis of groups by chronological age, biological age, and EAA/EAD.

Group	n	Chronological Age, y		Predicted Age, y		Statistical Significance of Differences Between Chronological and Predicted Age *p*	EAA/EAD (Differences Between Actual and Predicted Ages)	
		Median	p *	Median	*p*		Median	*p*
Group I Group II Group III Group IV	7 16 29 12	33.00 33.00 35.00 35.50	0.73	32.80 33.35 33.30 33.50	0.76	0.67 0.53 0.30 0.56	0.00 −0.15 0.60 0.10	0.99
ART: yes no	41 23	35.00 33.00	0.28	33.30 33.20	0.33	0.19 0.37	0.50 −0.10	0.86
BMI, kg/m^2^: normal deviation	48 16	35.00 33.50	0.31	33.40 32.50	0.19	0.31 0.19	0.40 0.20	0.54
AMH, ng/mL: >1.2 <1.2	47 17	33.00 35.00	0.19	33.20 33.30	0.26	0.47 0.08	−0.10 1.70	0.35

* Kruskal–Wallis/Wilcoxon signed-rank test.

**Table 6:** Characteristics of the analyzed age-related candidate DNA methylation loci.

Locus	Gene Name	Gene Function	CpG Sites	Chromosome Location (GRCh38)
*KLF14*	Kruppel-like factor 14	Transcription factor	C1	Chr7: 130734355
*FHL2*	Four and a half LIM domains protein 2	Transcription factor	C2	Chr2: 105399288
*TRIM59*	Tripartite motif containing 59	Regulator of immune signaling pathways	C7	Chr3: 160450199
*C1orf132*	-	-	C1	Chr1: 207823681
*ELOVL2*	ELOVL fatty acid elongase 2	Synthesis of very long chain polyunsaturated fatty acids	C5	Chr6: 11044875
			C7	Chr6: 11044867

## Data Availability

The original contributions presented in this study are included in the article and Supplementary Materials. Further inquiries can be directed to the corresponding author.

## Supplementary Materials

The following supporting information can be downloaded at: https://www.mdpi.com/article/10.3390/epigenomes9020019/s1, Figure S1: Differences in DNA methylation of *KLF14* C1, *FHL2* C2, *TRIM59* C7, *C1orf132* C1, *ELOVL2* C5 and *ELOVL2* C7 between age groups in the training group; Table S1: List of PCR primers, pyrosequencing primers, and sequences used in analysis; Table S2: DNA methylation levels in a sample of healthy women and women with perinatal losses and infertility; Table S3: DNA methylation levels in the training group.

## Abbreviations

The following abbreviations are used in this manuscript:

EAA	epigenetic age acceleration
EAD	epigenetic age deceleration
AMH	anti-Müllerian hormone
FSH	follicle-stimulating hormone
E2	estradiol
AFC	antral follicle count
ART	assisted reproductive technology
BMI	body mass index
RIF	recurrent implantation failure
MAD	mean absolute deviation
MAE	mean absolute error
RMSE	root mean squared error
SEE	standard error of the estimate
SE	standard error
SD	standard deviation

## References

[1] Datta J., Palmer M.J., Tanton C., Gibson L.J., Jones K.G., Macdowall W., Glasier A., Sonnenberg P., Field N., Mercer C.H. (2016). Prevalence of infertility and help seeking among 15 000 women and men. Hum. Reprod..

[2] Boedt T., Vanhove A.C., Vercoe M.A., Matthys C., Dancet E., Fong S.L. (2021). Preconception lifestyle advice for people with infertility. Cochrane Database Syst. Rev..

[3] Hernaez A., Rogne T., Skara K.H., Haberg S.E., Page C.M., Fraser A., Burgess S., Lawlor D.A., Magnus M.C. (2021). Body mass index and subfertility: Multivariable regression and Mendelian randomization analyses in the Norwegian Mother, Father and Child Cohort Study. Hum. Reprod..

[4] Hernaez A., Wootton R.E., Page C.M., Skara K.H., Fraser A., Rogne T., Magnus P., Njolstad P.R., Andreassen O.A., Burgess S. (2022). Smoking and infertility: Multivariable regression and Mendelian randomization analyses in the Norwegian Mother, Father and Child Cohort Study. Fertil. Steril..

[5] ESHRE Capri Workshop Group (2017). A prognosis-based approach to infertility: Understanding the role of time. Hum. Reprod..

[6] Bell C.G., Lowe R., Adams P.D., Baccarelli A.A., Beck S., Bell J.T., Christensen B.C., Gladyshev V.N., Heijmans B.T., Horvath S. (2019). DNA methylation aging clocks: Challenges and recommendations. Genome Biol..

[7] Chen B.H., Marioni R.E., Colicino E., Peters M.J., Ward-Caviness C.K., Tsai P.C., Roetker N.S., Just A.C., Demerath E.W., Guan W. (2016). DNA methylation-based measures of biological age: Meta-analysis predicting time to death. Aging.

[8] Horvath SRaj K. (2018). DNA methylation-based biomarkers and the epigenetic clock theory of aging. Nat. Rev.Genet..

[9] Horvath S. (2013). DNA methylation age of human tissues and cell types. Genome Biol..

[10] Zbiec-Piekarska R., Spolnicka M., Kupiec T., Makowska Z., Spas A., Parys-Proszek A., Kucharczyk K., Ploski P., Wojciech Branicki W. (2015). Examination of DNA methylation status of the ELOVL2 marker may be useful for human age prediction in forensic science. Forensic Sci. Int. Genet..

[11] Zbiec-Piekarska R., Spolnicka M., Kupiec T., Parys-Proszek A., Makowska Z., Pałeczka A., Kucharczyk K., Płoski R., Branicki W. (2015). Development of a forensically useful age prediction method based on DNA methylation analysis. Forensic Sci. Int. Genet..

[12] Park J.L., Kim J.H., Seo E., Bae D.H., Kim S.Y., Lee H.C., Woo K.M., Kim Y.S. (2016). Identification and evaluation of age-correlated DNA methylation markers for forensic use. Forensic Sci. Int. Genet..

[13] Cho S., Jung S.-E., Hong S.R., Lee E.H., Lee J.H., Lee S.D., Lee H.Y. (2017). Independent validation of DNA-based approaches for age prediction in blood. Forensic Sci. Int. Genet..

[14] Spólnicka M., Pośpiech E., Pepłońska B., Zbieć-Piekarska R., Makowska Ż., Pięta A., Karłowska-Pik J., Ziemkiewicz B., Wężyk M., Gasperowicz P. (2018). DNA methylation in ELOVL2 and C1orf132 correctly predicted chronological age of individuals from three disease groups. Int. J. Legal. Med..

[15] Spólnicka M., Pośpiech E., Adamczyk J.G., Freire-Aradas A., Pepłońska B., Zbieć-Piekarska R., Makowska Ż., Pięta A., Lareu M.V., Phillips C. (2018). Modified aging of elite athletes revealed by analysis of epigenetic age markers. Aging.

[16] Marcante B., Marino L., Cattaneo N.E., Delicati A., Tozzo P., Caenazzo L. (2025). Advancing forensic human chronological age estimation: Biochemical, genetic, and epigenetic approaches from the last 15 years: A systematic review. Int. J. Mol. Sci..

[17] Piani L.L., Vigano P., Somigliana E. (2023). Epigenetic clocks and female fertility timeline: A new approach to one an old issue?. Front. Cell Dev. Biol..

[18] Shuster L.T., Gostout B.S., Grossardt B.R., Rocca W.A. (2008). Prophylactic oophorectomy in premenopausal women and long-term health. Menopause Int..

[19] Mason J.B., Cargill S.L., Anderson G.B., Carey J.R. (2009). Transplantation of young ovaries to old mice increased life span in transplant recipients. J. Gerontol. A Biol. Sci. Med. Sci..

[20] Levine M.E., Lu A.T., Chen B.H., Hernandez D.G., Singleton A.B., Ferrucci L., Bandinelli S., Salfati E., Manson J.E., Quach A. (2016). Menopause accelerates biological aging. Proc. Natl. Acad. Sci. USA.

[21] Xie W., Li L., Zheng X.L., Yin W.D., Tang C.K. (2017). The role of Krüppel-like factor 14 in the pathogenesis of atherosclerosis. Atherosclerosis.

[22] Wu S., Wu S., Hsu L.A., Teng M.S., Chou H.H., Ko Y.L. (2022). Differential genetic and epigenetic effects of the KLF14 gene on body shape indices and metabolic traits. Int. J. Mol. Sci..

[23] Koppes E., Shaffer B., Sadovsky E., Himes K., Barak Y., Sadovsky Y., Chaillet J.R. (2019). Klf14 is an imprinted transcription factor that regulates placental growth. Placenta.

[24] Slieker R.C., Relton C.L., Gaunt T.R., Slagboom P.E., Heijmans B.T. (2018). Age-related DNA methylation changes are tissue-specific with ELOVL2 promoter methylation as exception. Epigenet. Chromatin..

[25] Zhu T., Zheng S.C., Paul D.S., Horvath S., Teschendorff A.E. (2018). Cell and tissue type independent age-associated DNA methylation changes are not rare but common. Aging.

[26] Ibáñez-Cabellos J.S., Sandoval J., Pallardó F.V., García-Giménez J.L., Mena-Molla S. (2025). A sex-specific minimal CpG-based model for biological aging using ELOVL2 methylation analysis. Int. J. Mol. Sci..

[27] Chen D., Chao D.L., Rocha L., Kolar M., Nguyen Huu V.A., Krawczyk M., Dasyani M., Wang T., Jafari M., Jabari M. (2020). The lipid elongation enzyme ELOVL2 is a molecular regulator of aging in the retina. Aging Cell.

[28] Chao D.L., Skowronska-Krawczyk D. (2020). ELOVL2: Not just a biomarker of aging. Transl. Med. Aging.

[29] Durso D.F., Bacalini M.G., Sala C., Pirazzini C., Marasco E., Bonafé M., do Valle Í.F., Gentilini D., Castellani G., Faria A.M.C. (2017). Acceleration of leukocytes’ epigenetic age as an early tumor and sex-specific marker of breast and colorectal cancer. Oncotarget.

[30] González R.S., Rodriguez-Cruz M., Maldonado J., Saavedra F.J. (2014). Role of maternal tissue in the synthesis of polyunsaturated fatty acids in response to a lipid-deficient diet during pregnancy and lactation in rats. Gene.

[31] Jung S.E., Lim S.M., Hong S.R., Lee E.H., Shin K.J., Lee H.Y. (2019). DNA methylation of the ELOVL2, FHL2, KLF14, C1orf132/MIR29B2C, and TRIM59 genes for age prediction from blood, saliva, and buccal swab samples. Forensic Sci. Int. Genet..

[32] Lee H.Y., Hong S.R., Lee J.E., Hwang I.K., Kim N.Y., Lee J.M., Fleckhaus J., Jung S.E., Lee Y.H. (2020). Epigenetic age signatures in bones. Forensic Sci. Int. Genet..

[33] Habibe J.J., Clemente-Olivo M.P., Vries C.J. (2021). How (Epi)genetic regulation of the LIM-domain protein FHL2 impacts multifactorial disease. Cells.

[34] Bae H., Lunetta K.L., Murabito J.M., Andersen S.L., Schupf N., Perls T., Sebastiani P. (2019). Genetic associations with age of menopause in familial longevity. Menopause.

[35] Tachmazidou I., Süveges D., Min J.L., Ritchie G.R.S., Steinberg J., Walter K., Iotchkova V., Schwartzentruber J., Huang J., Memari Y. (2017). Whole-genome sequencing coupled to imputation discovers genetic signals for anthropometric traits. Am. J. Hum. Genet..

[36] Zhu Z., Guo Y., Shi H., Liu C.L., Panganiban R.A., Chung W., O’Connor L.J., Himes B.E., Gazal S., Hasegawa K. (2020). Shared genetic and experimental links between obesity-related traits and asthma subtypes in UK Biobank. J. Allergy Clin. Immunol..

[37] Shafaroudi A.M., Sharifi-Zarchi A., Saeid Rahmani S., Nafissi N., Mowla S.J., Lauria A., Oliviero S., Matin M.M. (2021). Expression and function of C1orf132 long-noncoding RNA in breast cancer cell lines and tissues. Int. J. Mol. Sci..

[38] Jin Z., Liu L., Yu Y., Li D., Zhu X., Yan D., Zhu Z. (2021). TRIM59: A potential diagnostic and prognostic biomarker in human tumors. PLoS ONE.

[39] Wang F., Wang H., Sun L., Niu C., Xu J. (2020). TRIM59 inhibits PPM1A through ubiquitination and activates TGF-β/Smad signaling to promote the invasion of ectopic endometrial stromal cells in endometriosis. Am. J. Physiol. Cell Physiol..

[40] Wu T., Zhou H., Wang L., Tan J., Gao W., Wu Y., Zhao D., Shen C., Zheng B., Huang X. (2024). TRIM59 is required for mouse GC-1 cell maintenance through modulating the ubiquitination of AXIN1. Heliyon.

[41] Frank E., Hall M.A., Witten I.H. (2016). The WEKA Workbench. Online Appendix for “Data Mining: Practical Machine Learning Tools and Techniques”.

[42] Fritz R., Jindal S. (2018). Reproductive aging and elective fertility preservation. J. Ovarian Res..

[43] Cil A.P., Turkgeldi L., Seli E. (2015). Oocyte cryopreservation as a preventive measure for age-related fertility loss. Semin. Reprod. Med..

[44] Daunay A., Baudrin L.G., Deleuze J.F., How-Kit A. (2019). Evaluation of six blood-based age prediction models using DNA methylation analysis by pyrosequencing. Sci. Rep..

[45] Thong Z., Liang Shun Chan X., Ying Ying Tan J., Shuzhen Loo E., Kiu Choong Syn C. (2017). Evaluation of DNA methylation-based age prediction on blood. Forensic Sci. Int. Genet. Suppl. Ser..

[46] Hanson B.M., Tao X., Zhan Y., Jenkins T.G., Morin S.J., Scott R.T., Seli E.U. (2020). Young women with poor ovarian response exhibit epigenetic age acceleration based on evaluation of white blood cells using a DNA methylation-derived age prediction model. Hum. Reprod..

[47] Monseur B., Murugappan G., Bentley J., Teng N., Westphal L. (2020). Epigenetic clock measuring age acceleration via DNA methylation levels in blood is associated with decreased oocyte yield. J. Assist. Reprod. Genet..

[48] Tal R., Seifer D.B. (2017). Ovarian reserve testing: A user’s guide. Am. J. Obstet. Gynecol..

[49] Dogan M.V., Xiang J., Beach S.R.H., Cutrona C., Gibbons F.X., Simons R.L., Brody G.H., Stapleton J.T., Philibert R.A. (2015). Ethnicity and smoking-associated DNA methilation changes at HIV co-receptor GPR15. Front. Pshychiatry.

[50] Lei M.K., Gibbons F.X., Simons R.L., Philibert R.A., Beach S.R.H. (2020). The Effect of Tobacco Smoking Differs across Indices of DNA Methylation-Based Aging in an African American Sample: DNA Methylation-Based Indices of Smoking Capture These Effects. Genes.

[51] Philibert R., Beach S.R.H., Lei M.K., Gibbons F.X., Gerrard M., Simons R.L., Dogan M.V. (2020). Array-Based Epigenetic Aging Indices May Be Racially Biased. Genes.

[52] Levine M.E., Lu A.T., Quach A., Chen B.H., Assimes T., Bandinelli S., Hou L., Baccarelli A., Stewart J., Li Y. (2018). An epigenetic biomarker of aging for lifespan and healthspan. Aging.

[53] Ling C., Rönn T. (2019). Epigenetics in Human Obesity and Type 2 Diabetes. Cell Metab..

[54] Oblak L., van der Zaag J., Higgins-Chen A.T., Levine M.E., Boks M.P. (2021). A systematic review of biological, social and environmental factors associated with epigenetic clock acceleration. Ageing Res. Rev..

[55] Rodés B., Cadiñanos J., Esteban-Cantos A., Rodríguez-Centeno J., Ramón Arribas J. (2022). Ageing with HIV: Challenges and biomarkers. eBioMedicine.

[56] Ramaker M.E., Corcoran D.L., Apsley A.T., Kobor M.S., Kraus V.B., Kraus W.E., Lin D.T.S., Orenduff M.C., Pieper C.F., Waziry R. (2022). Epigenome-wide Association Study Analysis of Calorie Restriction in Humans, CALERIETM Trial Analysis. J. Gerontol. A. Biol. Sci. Med. Sci..

[57] Cao X., Li W., Wang T., Ran D., Davalos V., Planas-Serra L., Pujol A., Esteller M., Wang X., Yu H. (2022). Accelerated biological aging in COVID-19 patients. Nat. Commun..

[58] Moqri M., Herzog C., Poganik J.R., Ying K., Justice J.N., Belsky D.W., Higgins-Chen A.T., Chen B.H., Cohen A.A., Fuellen G. (2024). Validation of biomarkers of aging. Nat. Med..

[59] Hannum G., Guinney J., Zhao L., Zhang L., Hughes G., Sadda S., Klotzle B., Bibikova M., Fan J.B., Gao Y. (2013). Genome-wide methylation profiles reveal quantitative views of human aging rates. Mol. Cell..

[60] Lu A.T., Quach A., Wilson J.G., Reiner A.P., Aviv A., Raj K., Hou L., Baccarelli A.A., Li Y., Stewart J.D. (2019). DNA methylation GrimA.e strongly predicts lifespan and healthspan. Aging.

[61] Krolevets M., Cate V.T., Prochaska J.H., Schulz A., Rapp S., Tenzer S., Andrade-Navarro M.A., Horvath S., Niehrs C., Wild P.S. (2023). DNA methylation and cardiovascular disease in humans: A systematic review and database of known CpG methylation sites. Clin. Epigenetics.

[62] Li A., Koch Z., Ideker T. (2022). Epigenetic aging: Biological age prediction and informing amechanistic theory of aging. J. Intern. Med..

[63] https://www.r-project.org/.

[64] Wickham H. (2016). ggplot2. Elegant Graphics for Data Analysis.

[65] Patil I. (2021). Visualizations with statistical details: The ‘ggstatsplot’ approach. J. Open Source Softw..

[66] Lüdecke D., Ben-Shachar M.S., Patil I., Waggoner P., Makowski D. (2021). Performance: An R Package for Assessment, Comparison and Testing of Statistical Models. J. Open Source Softw..

