# Supplementary material for: Association of Model-Predicted Epigenetic Age and Female Infertility
Source: Epigenomes. 2025 Jun 5;9(2):19. doi: 10.3390/epigenomes9020019 (PMC12192080; doi:10.3390/epigenomes9020019)
Supplement: Supplementary file 1 [file epigenomes-09-00019-s001.zip › Suppl. Figure S1.pdf]

Figure S1. Differences in DNA methylation of (**a**) *KLF14* C1, (**b**) *FHL2* C2, (**c**) *TRIM59* C7, (**d**) *C1orf132* C1, (**e**) *ELOVL2* C5 and (**f**) *ELOVL2* C7 between age groups in the training group.

(a)

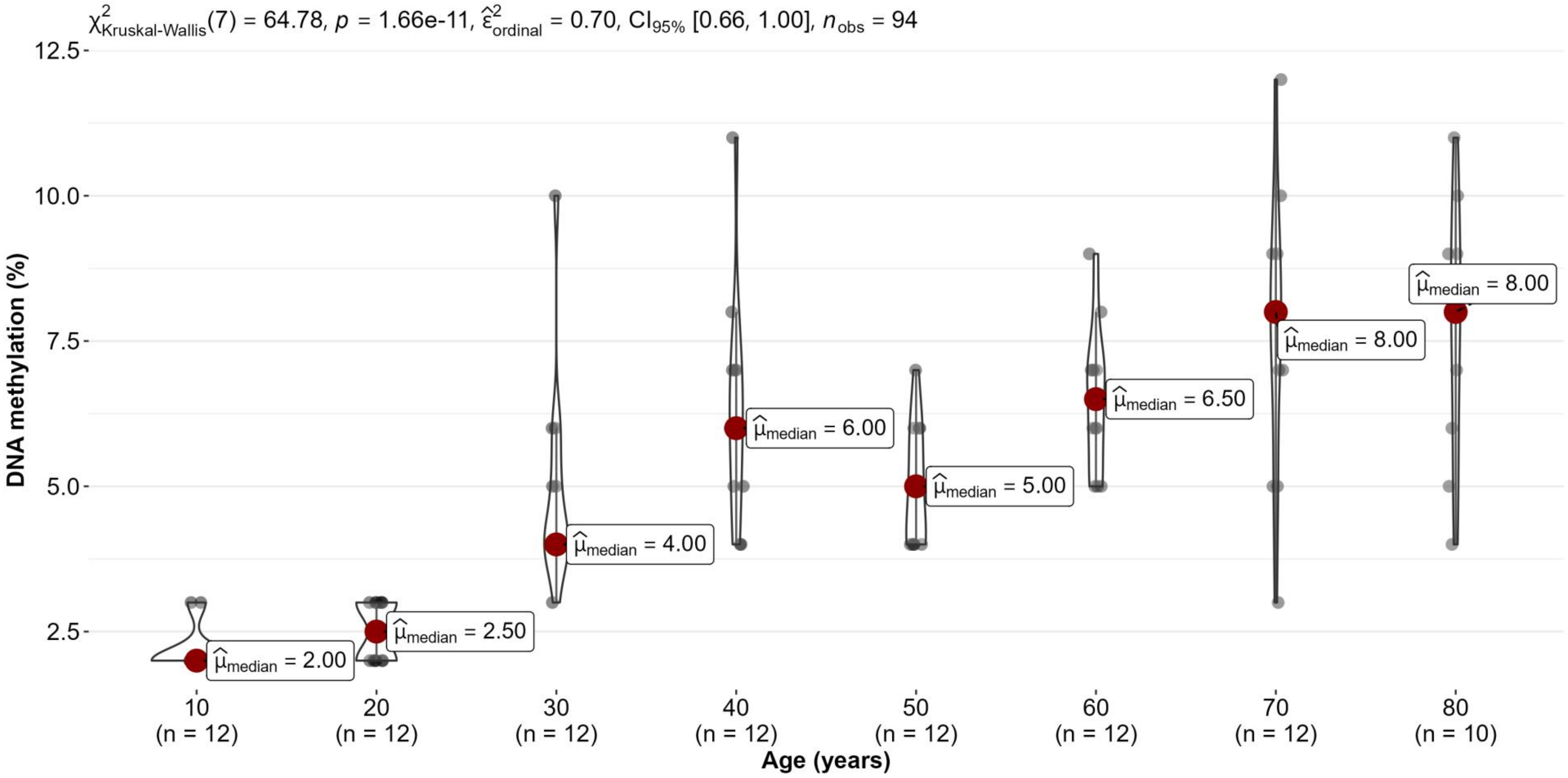

(b)

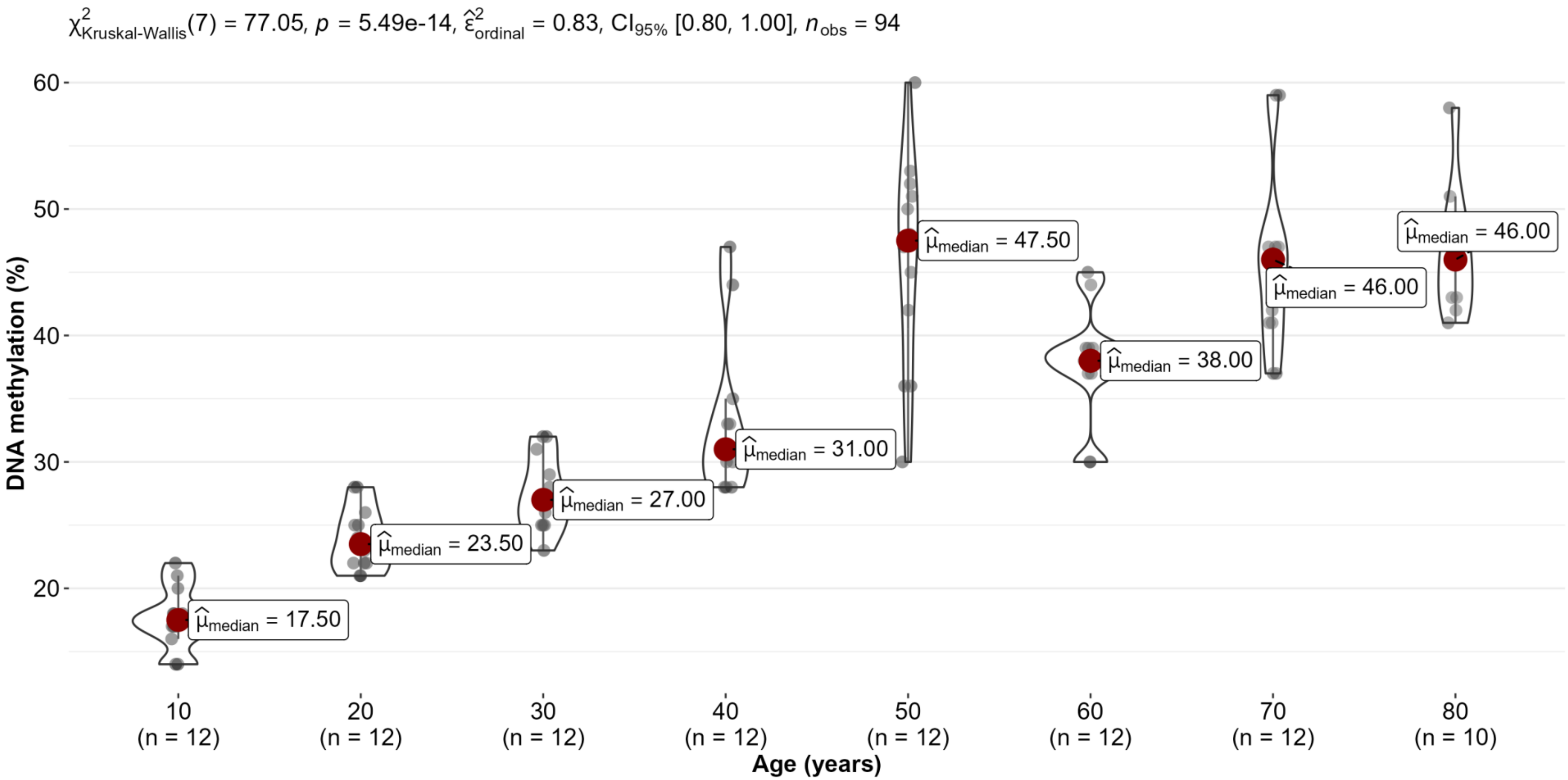

χ²Kruskal-Wallis(7) = 77.05, p = 5.49e-14, ε̂²ordinal = 0.83, CI95% [0.80, 1.00], nobs = 94
DNA methylation (%)
60
50
40
30
20
μ̂median = 17.50
μ̂median = 23.50
μ̂median = 27.00
μ̂median = 31.00
μ̂median = 47.50
μ̂median = 38.00
μ̂median = 46.00
μ̂median = 46.00
10 (n = 12)
20 (n = 12)
30 (n = 12)
40 (n = 12)
50 (n = 12)
60 (n = 12)
70 (n = 12)
80 (n = 10)
Age (years)

(c)

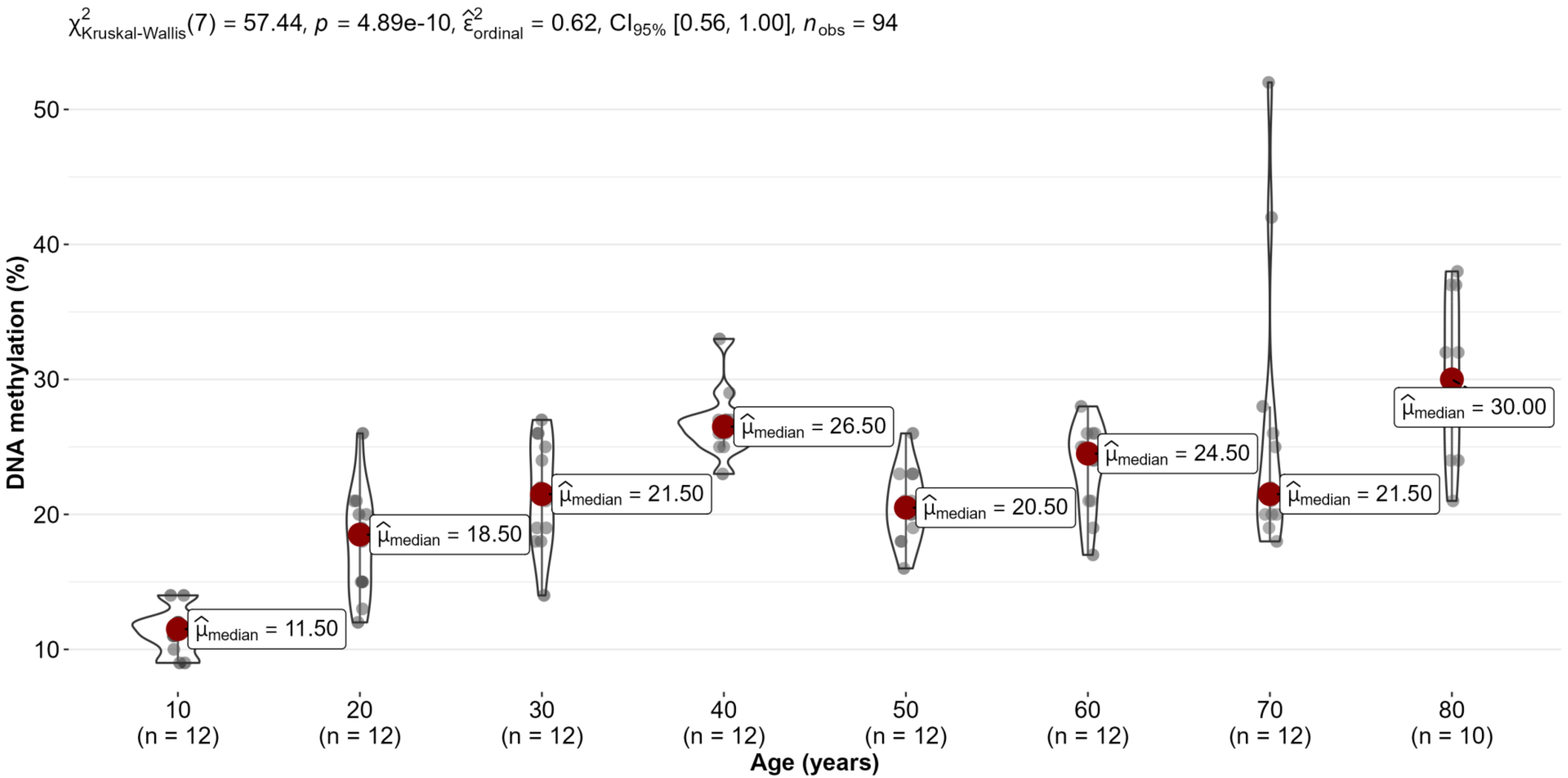
χ²Kruskal-Wallis(7) = 57.44, p = 4.89e-10, ε̂²ordinal = 0.62, CI95% [0.56, 1.00], nobs = 94
DNA methylation (%)
10
20
30
40
50
μ̂median = 11.50
μ̂median = 18.50
μ̂median = 21.50
μ̂median = 26.50
μ̂median = 20.50
μ̂median = 24.50
μ̂median = 21.50
μ̂median = 30.00
10 (n = 12)
20 (n = 12)
30 (n = 12)
40 (n = 12)
50 (n = 12)
60 (n = 12)
70 (n = 12)
80 (n = 10)
Age (years)

(d)

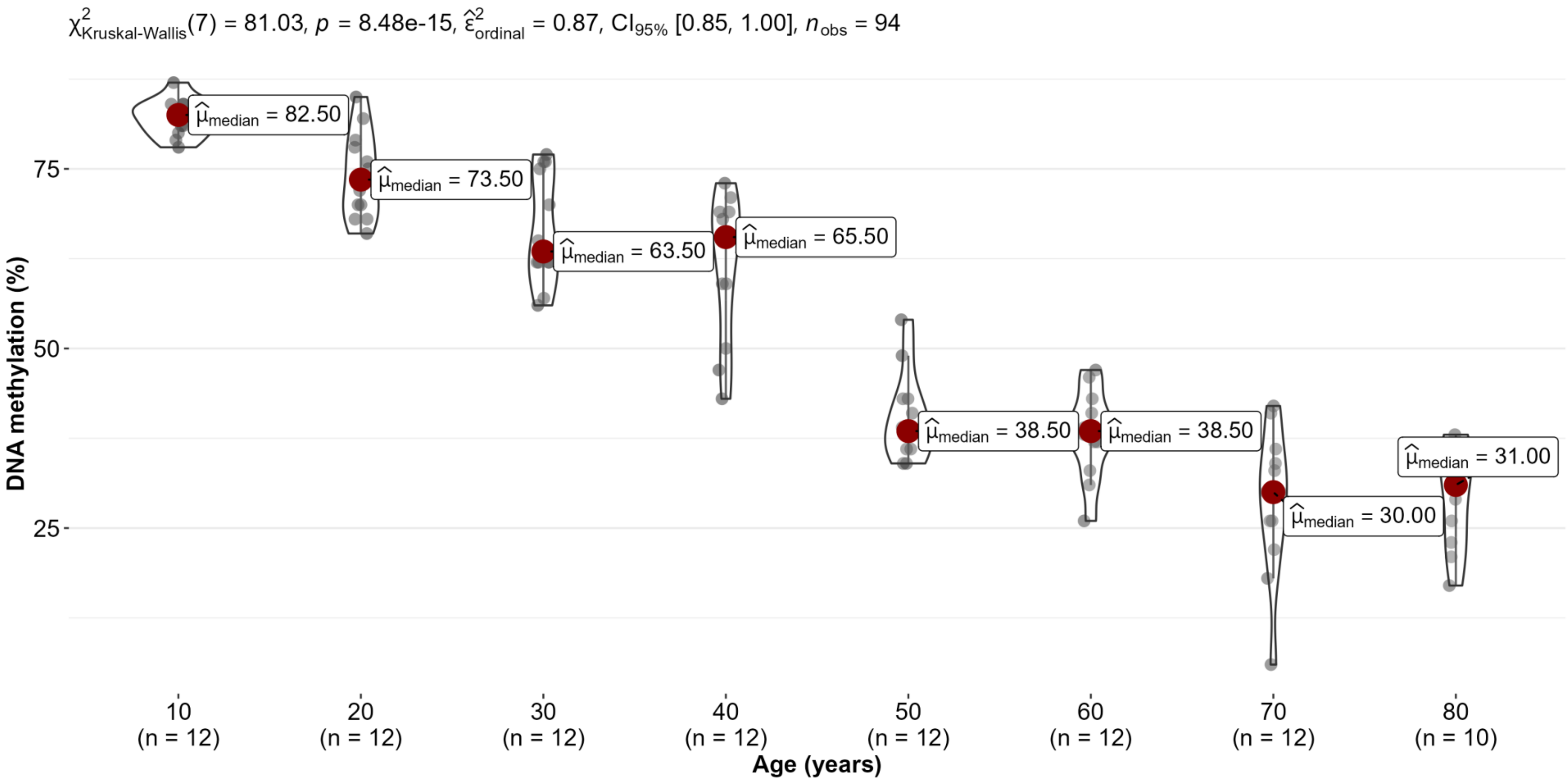

$\chi^2_{\text{Kruskal-Wallis}}(7) = 81.03$, $p = 8.48e-15$, $\hat{\varepsilon}^2_{\text{ordinal}} = 0.87$, $CI_{95\%}$ [0.85, 1.00], $n_{obs} = 94$
$\hat{\mu}_{median} = 82.50$
$\hat{\mu}_{median} = 73.50$
$\hat{\mu}_{median} = 63.50$
$\hat{\mu}_{median} = 65.50$
$\hat{\mu}_{median} = 38.50$
$\hat{\mu}_{median} = 38.50$
$\hat{\mu}_{median} = 30.00$
$\hat{\mu}_{median} = 31.00$
DNA methylation (%)
75
50
25
10 (n = 12)
20 (n = 12)
30 (n = 12)
40 (n = 12)
50 (n = 12)
60 (n = 12)
70 (n = 12)
80 (n = 10)
Age (years)

(e)

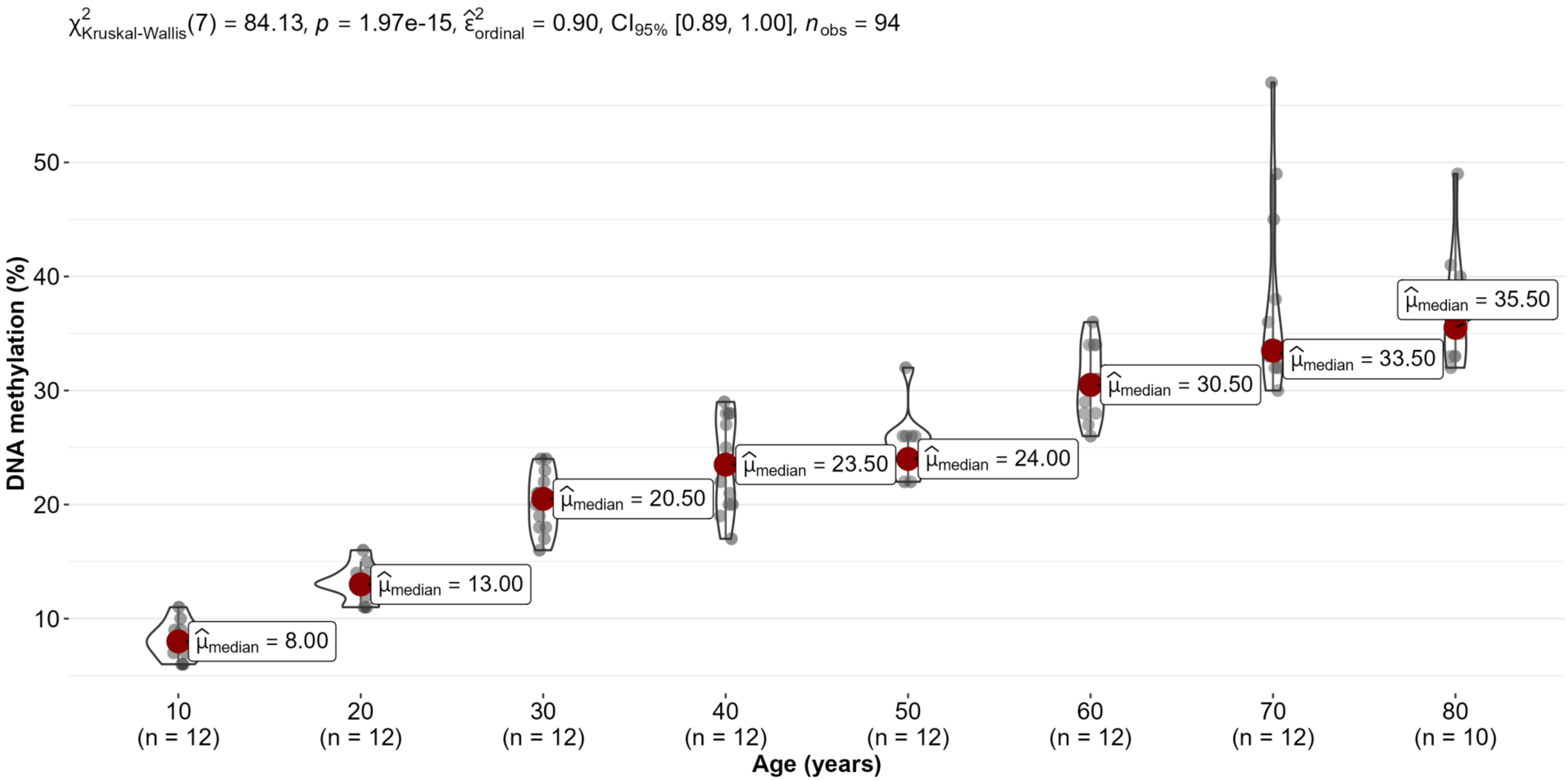

$\chi^2_{\text{Kruskal-Wallis}}(7) = 84.13$, $p = 1.97\text{e-}15$, $\hat{\varepsilon}^2_{\text{ordinal}} = 0.90$, $\text{CI}_{95\%}$ [0.89, 1.00], $n_{\text{obs}} = 94$
$\hat{\mu}_{\text{median}} = 8.00$
$\hat{\mu}_{\text{median}} = 13.00$
$\hat{\mu}_{\text{median}} = 20.50$
$\hat{\mu}_{\text{median}} = 23.50$
$\hat{\mu}_{\text{median}} = 24.00$
$\hat{\mu}_{\text{median}} = 30.50$
$\hat{\mu}_{\text{median}} = 33.50$
$\hat{\mu}_{\text{median}} = 35.50$
DNA methylation (%)
10
20
30
40
50
10 (n = 12)
20 (n = 12)
30 (n = 12)
40 (n = 12)
50 (n = 12)
60 (n = 12)
70 (n = 12)
80 (n = 10)
Age (years)

(f)

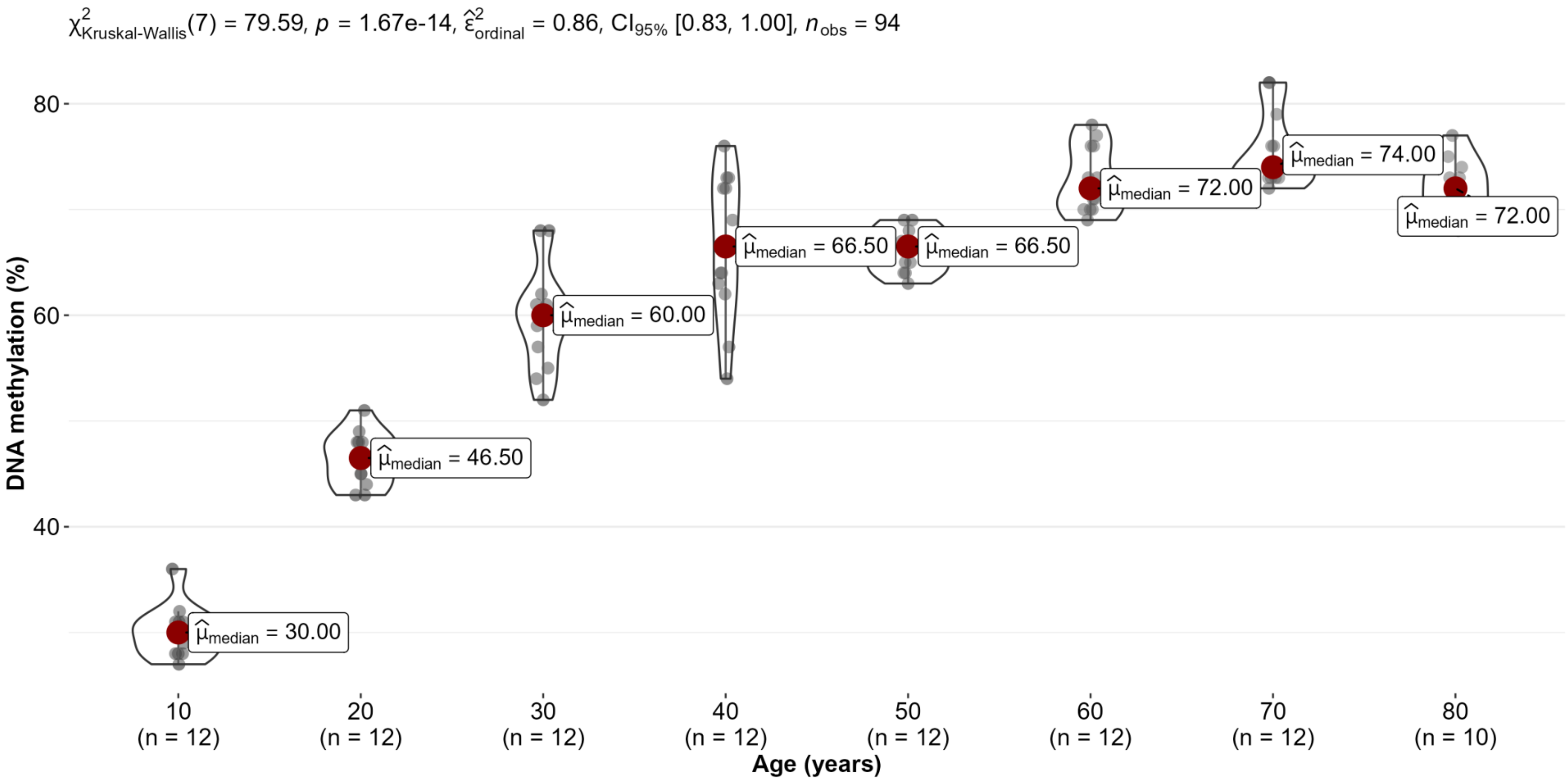
$\chi^2_{Kruskal-Wallis}(7) = 79.59$, $p = 1.67e-14$, $\hat{\varepsilon}^2_{ordinal} = 0.86$, $CI_{95\%}$ [0.83, 1.00], $n_{obs} = 94$
DNA methylation (%)
80
60
40
$\hat{\mu}_{median} = 30.00$
$\hat{\mu}_{median} = 46.50$
$\hat{\mu}_{median} = 60.00$
$\hat{\mu}_{median} = 66.50$
$\hat{\mu}_{median} = 66.50$
$\hat{\mu}_{median} = 72.00$
$\hat{\mu}_{median} = 74.00$
$\hat{\mu}_{median} = 72.00$
10 (n = 12)
20 (n = 12)
30 (n = 12)
40 (n = 12)
50 (n = 12)
60 (n = 12)
70 (n = 12)
80 (n = 10)
Age (years)
