# Supplementary material for: Association of Model-Predicted Epigenetic Age and Female Infertility
Source: Epigenomes. 2025 Jun 5;9(2):19. doi: 10.3390/epigenomes9020019 (PMC12192080; doi:10.3390/epigenomes9020019)
Supplement: Supplementary file 1 [file epigenomes-09-00019-s001.zip › Suppl. Table S1.pdf]

Supplementary Table S1. List of PCR primers, pyrosequencing primers and sequences for analysis

Primer	Primer description	Primer sequence, 5′-3′	Sequence to analyze
ELOVL2-F	PCR_Forward	TAGGTTTAGGGGGTTGGGTGGGTTT	
ELOVL2-R	PCR_Reverse	biotin-GAGCGGGACAGAGACTCTTGCTCA	CCRAAACRTTAAACCRCCRCRCRAAACCRAC
ELOVL2-S	Pyrosequencing	GGTTGGGTGGGTTTCTCTGG	
KLF14-F	PCR_Forward	GGTTTTTAGGTTAAGTTATGTTTAATAGT	
KLF14-R	PCR_Reverse	biotin-AAACTACTACAACCCAAAAAATTCC	TYGYGTTTTTTTTTTTGTYGGYGAGTTAGGTA
KLF14-S	Pyrosequencing	ATAGTTTTAGAAATTATTTTGTTT	
FHL2-F	PCR_Forward	GGGTTTTGGGAGTATAGTAGT	
FHL2-R	PCR_Reverse	biotin-TCTCCCCCTCCTAAAACCAAACAAAAATC	AGTTATYGGGAGYGTYGTTTTYGGYGTGGGTTTTYG<br>GGYGYGAGTTTYGGAYGAGGTTTGGG
FHL2-S	Pyrosequencing	GGGTTTTGGGAGTATAGT	
Orf132-F	PCR_Forward	biotin-AAGAAGGTGAGAAAGATAGAGTAT	
Orf132-R	PCR_Reverse	ATTTAATAAAACCAAATTCTAAAACATTC	AAATCTACRCAAACRACRATAAATAATCC
Orf132-S	Pyrosequencing	ACACCTTACCACCAAACCAAAATTT	
TRIM59-F	PCR_Forward	TATAGGTGGTTTGGGGGAGAG	
TRIM59-R	PCR_Reverse	biotin-AAAAAACACTACCCTCCACAACATAAC	GGTTTGGYGYGGGAYGAGGYGAAGYGTYGGTGGTY<br>GAYGGTTTTTGAGGAATTATTTTTTATTT
TRIM59-S	Pyrosequencing	GGTGGTTTGGGGGAGAGGTTG	
