# Supplementary material for: Association of Model-Predicted Epigenetic Age and Female Infertility
Source: Epigenomes. 2025 Jun 5;9(2):19. doi: 10.3390/epigenomes9020019 (PMC12192080; doi:10.3390/epigenomes9020019)
Supplement: Supplementary file 1 [file epigenomes-09-00019-s001.zip › Suppl. Table S2.pdf]

Supplementary Table S2. DNA methylation levels in a sample of healthy women and women with perinatal losses and infertility

Sample ID	Group	BMI	ART	Chronological age	AMH, ng/ml	*C1orf132*, %	*ELOVL2*_CpG5, %	*ELOVL2*_CpG7, %	*FHL2*, %	*TRIM59*, %	*KLF14*, %
704	Group III	20,96	yes	24	2,15	66	16	51	26	15	3
780	Group III	18,78	yes	27	2,46	63	16	53	29	18	3
772	Group I	17,36	no	28	1,76	65	16	54	28	22	4
812	Group II	18,65	no	28	4,68	72	18	57	32	19	3
822	Group II	19,35	no	29	4,2	64	16	55	31	23	3
887	Group III	19,27	yes	29	3,4	68	18	55	32	24	3
727	Group II	19,32	no	30	3,67	72	18	54	29	21	4
750	Group III	21,56	yes	30	2,35	61	19	59	32	22	4
863	Group II	19,98	no	30	2,1	72	20	56	33	21	6
864	Group II	23,78	no	30	4,68	64	20	59	35	24	4
765	Group III	27,34	yes	31	4,25	68	21	60	27	23	4
771	Group IV	17,99	yes	31	4,65	64	18	58	30	21	4
840	Group III	19,76	yes	31	1,82	64	19	57	33	24	3
852	Group I	24,38	no	31	7,93	74	21	61	34	28	5
751	Group II	19,43	no	32	18,15	61	23	55	31	29	3
781	Group III	20,75	yes	32	3,09	67	18	59	29	21	4
788	Group I	21,88	no	32	3,7	64	21	61	32	20	5
801	Group III	20,75	yes	32	3,09	65	19	51	29	22	4
836	Group II	20	no	32	2,28	72	18	53	36	29	4
868	Group III	25,06	yes	32	1,3	76	18	59	36	25	4
870	Group III	20,73	yes	32	1,89	54	18	59	37	15	4
7801	Group II	20,48	no	32	3,89	66	18	55	22	21	4
7802	Group IV	32,08	yes	32	2,87	59	18	55	30	18	3
811	Group I	17,7	no	33	10,38	62	16	54	32	27	3
737	Group I	17,85	no	34	6,38	62	18	58	35	28	3
775	Group III	23,31	yes	34	2,54	57	18	61	29	22	5

885	Group II	21,68	no	34	2,74	69	19	58	31	25	4
776	Group II	20,96	no	35	2	67	21	64	34	24	5
753	Group II	19,13	no	36	1,79	65	21	61	33	25	5
754	Group III	29,86	yes	36	2,14	66	22	62	34	20	4
758	Group III	19,49	yes	36	6,89	66	21	62	36	24	5
774	Group III	21,3	yes	36	2,14	60	22	65	35	26	4
867	Group IV	27,76	yes	36	1,43	63	21	64	35	23	5
890	Group III	28,48	yes	36	3,45	56	17	59	40	21	4
894	Group III	19,71	yes	36	1,83	69	22	61	35	19	4
716	Group III	17,87	yes	37	2,14	59	21	63	28	20	5
729	Group I	19,82	no	37	3,2	57	21	63	32	23	4
778	Group II	20,08	no	37	1,62	59	23	64	31	28	4
769	Group I	26,87	no	38	2,75	55	20	56	32	22	5
803	Group II	21,91	no	38	1,67	56	20	62	36	23	6
820	Group IV	20,86	yes	38	1,61	65	25	66	38	19	4
835	Group III	29,65	yes	38	1,88	63	19	63	37	30	5
838	Group IV	18,38	yes	38	2,02	70	21	60	35	24	8
841	Group IV	23,71	yes	38	2,01	65	23	66	33	21	6
842	Group III	19,43	yes	38	4,7	69	22	62	31	24	5
733	Group III	24	yes	39	3,14	44	23	65	33	17	4
877	Group II	20,45	no	39	3,16	53	20	63	40	28	5
736	Group IV	19,35	yes	30	0,09	64	19	57	31	21	4
846	Group IV	19,65	yes	30	0,35	57	16	54	28	19	4
741	Group IV	22,43	yes	34	0,34	68	20	60	27	23	4
866	Group III	23,05	yes	34	0,87	70	21	61	32	25	4
732	Group IV	19,1	yes	35	0,78	60	21	61	33	27	4
740	Group III	21,36	yes	35	0,91	59	19	59	32	17	4
782	Group III	22,68	yes	35	0,6	64	18	59	31	22	5
787	Group III	19,95	yes	35	0,36	54	21	61	28	22	5

793	Group III	19,1	yes	35	0,19	73	20	61	38	24	4
843	Group III	21,39	yes	35	0,3	65	20	60	34	30	4
701	Group II	22,04	no	36	1,19	64	20	61	32	24	5
730	Group III	19,71	yes	36	1,1	59	19	56	31	24	3
757	Group IV	20,41	yes	36	0,64	68	22	62	33	24	4
869	Group II	23,53	no	36	0,36	59	23	62	31	20	5
826	Group IV	20,08	yes	37	0,09	59	21	63	32	25	4
752	Group III	19,72	yes	39	0,75	57	23	62	37	23	4
901	Group III	20,02	yes	39	0,2	67	22	63	36	23	9
