# Supplementary material for: Association of Model-Predicted Epigenetic Age and Female Infertility
Source: Epigenomes. 2025 Jun 5;9(2):19. doi: 10.3390/epigenomes9020019 (PMC12192080; doi:10.3390/epigenomes9020019)
Supplement: Supplementary file 1 [file epigenomes-09-00019-s001.zip › Suppl. Table S3.pdf]

Supplementary Table S3. DNA methylation levels in the training group

Sample ID	Chronological age	FHL2, %	KLF14, %	TRIM59, %	C1orf132, %	ELOVL2_CpG5, %	ELOVL2_CpG7, %
10_01	10	16	2	11	79	7	32
10_02	10	18	2	9	78	8	28
10_03	10	14	2	14	83	6	36
10_04	10	22	2	12	81	7	30
10_05	10	17	3	12	83	10	31
10_06	10	18	2	14	80	11	31
10_07	10	17	2	12	84	8	28
10_08	10	18	2	12	81	9	30
10_9	10	17	2	10	84	8	28
10_10	10	21	3	11	84	6	29
10_11	10	14	2	9	87	9	31
10_12	10	20	2	11	82	8	27
20_01	20	22	3	13	66	15	43
20_02	20	24	3	15	68	11	43
20_03	20	26	2	20	82	13	48
20_04	20	21	3	15	79	16	51
20_05	20	25	2	21	70	13	46
20_06	20	21	3	15	76	13	44
20_07	20	25	2	12	70	12	45
20_08	20	23	3	18	75	14	48
20_09	20	28	2	19	72	11	48
20_10	20	22	2	21	68	14	49
20_11	20	22	2	26	85	13	45
20_12	20	28	3	20	78	13	47
30_01	30	29	4	21	62	18	55
30_02	30	28	5	27	76	23	61
30_03	30	32	6	26	77	24	68
30_04	30	26	6	25	70	21	60
30_05	30	27	4	26	62	24	68
30_06	30	25	4	19	62	19	54
30_07	30	32	4	18	56	21	60
30_08	30	27	10	22	75	22	61
30_09	30	25	4	18	65	17	57
30_10	30	31	3	19	62	18	59
30_11	30	23	5	24	76	20	62
30_12	30	25	4	14	57	16	52
40_01	40	33	6	27	73	25	73
40_02	40	28	8	27	68	28	76
40_03	40	47	5	33	69	28	72
40_04	40	35	6	26	65	21	62
40_05	40	31	7	23	59	19	54

40_06	40	44	6	29	71	29	72
40_07	40	30	7	25	69	28	73
40_08	40	33	11	27	43	20	64
40_09	40	28	4	26	59	20	63
40_10	40	31	7	27	66	27	69
40_11	40	30	5	25	47	22	64
40_12	40	28	4	26	50	17	57
50_01	50	52	6	23	34	26	69
50_02	50	48	6	20	36	26	67
50_03	50	47	4	18	36	24	65
50_04	50	50	5	19	38	24	64
50_05	50	53	4	21	38	22	63
50_06	50	60	4	26	34	32	68
50_07	50	51	6	16	39	22	69
50_08	50	36	7	18	54	24	65
50_09	50	36	4	23	49	24	67
50_10	50	30	5	21	41	26	64
50_11	50	42	4	20	43	24	66
50_12	50	45	5	23	43	26	67
60_01	60	39	7	25	37	34	76
60_02	60	38	7	24	43	27	70
60_03	60	37	6	24	38	34	77
60_04	60	38	6	26	37	28	70
60_05	60	38	8	26	47	30	73
60_06	60	39	9	26	31	34	76
60_07	60	30	5	21	39	29	70
60_08	60	37	5	25	39	31	73
60_09	60	44	6	17	33	28	71
60_10	60	45	7	28	46	26	69
60_11	60	39	7	21	26	36	78
60_12	60	30	5	19	41	31	71
70_01	70	46	5	18	22	38	74
70_02	70	46	9	19	36	30	73
70_03	70	37	8	20	30	33	72
70_04	70	42	8	20	26	36	74
70_05	70	41	9	22	41	33	73
70_06	70	47	12	28	42	49	73
70_07	70	59	7	52	6	57	82
70_08	70	59	3	42	30	45	82
70_09	70	41	5	25	18	32	79
70_10	70	47	8	26	33	34	76
70_11	70	37	7	20	26	32	73
70_12	70	47	10	21	34	32	76
80_01	80	58	4	37	36	49	73
80_02	80	51	6	28	17	41	75

80_03	80	43	11	32	38	33	69
80_04	80	43	7	32	33	35	73
80_05	80	46	9	38	26	38	77
80_06	80	41	9	21	23	36	71
80_07	80	49	5	24	34	33	70
80_08	80	47	8	37	21	40	74
80_09	80	46	10	24	35	33	71
80_10	80	42	8	28	29	32	68
